# The Impact of Work Connectivity Behavior on Employee Time Theft: The Role of Revenge Motive and Leader–Member Exchange

**DOI:** 10.3390/bs15060738

**Published:** 2025-05-27

**Authors:** Cuiying Wang, Jianfeng Huang, Jianping Zhu

**Affiliations:** School of Management, Xiamen University, Xiamen 361005, China; wang1967309094@outlook.com (C.W.);

**Keywords:** work connectivity behavior, employee time theft, revenge motive, leader–member exchange

## Abstract

Organizations have long been actively seeking ways to reduce unethical behavior among employees. However, employee time theft is widespread and costly across various industries, and related research remains relatively limited. Therefore, this study employed social exchange theory to empirically investigate how and when work connectivity behavior promotes employee time theft. Drawing on a sample of 330 employees, our findings indicate that work connectivity behavior positively impacts employee time theft by triggering revenge motives among employees. Furthermore, it was discovered that leader–member exchange weakens both the direct effect of work connectivity behavior on revenge motive and the indirect effect of work connectivity behavior on employee time theft via revenge motive. This research developed and elucidated a moderated mediation model, providing valuable insights for both theory and practice.

## 1. Introduction

The rapid advancement of information technology and the prevalence of the use of communication devices have significantly reorganized the temporal dynamics of the modern workplace (Reinke & Ohly, 2021). Extensive use of work-related communication tools outside work hours has become increasingly widespread (He et al., 2023). Repeated COVID-19 outbreaks over the past few years have compelled most firms to merge offline and online workspaces as part of their coping strategy and have led to the prevalence of the use of mobile devices and social media in organizational life (Liu et al., 2023; Stamm et al., 2023). Even outside the COVID-19 pandemic, the trend of remote work facilitated by devices such as computers and smartphones continues to rise (Chu et al., 2024). This development has contributed to work connectivity behavior (WCB), referred to as employees performing work-related tasks outside work hours through the use of mobile electronic devices and juggling multiple life and work roles (Brock et al., 2013). This “on call” expectation significantly impedes employees’ psychological detachment from work during off-hours (Jarvenpaa & Lang, 2005), leading to decreased well-being (Sonnentag et al., 2017) and increased stress (Sonnentag & Fritz, 2015), which in turn leads to diminished work engagement and performance in the workplace (Sonnentag, 2012). Whereas some literature highlights the benefits of WCB in the form of greater work autonomy and work flexibility (Y. Yang et al., 2023; Zhu et al., 2024), others suggest it damages family harmony (Dettmers et al., 2016) and mental and physical health (Dettmers et al., 2016), work attitudes, and job performance (Sonnentag & Niessen, 2020), and encourages online surfing (He et al., 2023) and unethical pro-family behavior (Liu et al., 2024).

However, while the previous literature has identified some negative effects of WCB, there is a lack of in-depth investigation into whether WCB will influence employee time theft. This oversight is unfortunate, as time theft can impose significant costs on organizations (Schmitt et al., 2003). In this study, employee time theft is defined as the deliberate misrepresentation and misappropriation of time spent on and paid by an organization (Harold et al., 2022). These comprise unauthorized breaks, work hour falsification, slow work rate, excessive socialization, and non-work activities (Harold et al., 2022). WCB, by encroaching on work in personal time and being undercompensated, leads employees to perceive a sense of inequality (Bennett & Robinson, 2000). Social exchange theory posits that perceived inequity leads employees to respond with similar negative behaviors (Kramer & Tyler, 1996; Van Jaarsveld et al., 2010). However, due to the power imbalance, employees often avoid directly retaliating against their supervisors and instead opt for more subtle methods to express their dissatisfaction, such as extending break times or engaging in non-work-related online activities during work hours (Lam & Xu, 2019). Therefore, this study posits that WCB may trigger employee time theft.

This study also examined how WCB contributes to employee time theft behavior. Revenge motive is defined as the intention of the victim of harm to inflict damage, injury, discomfort, or punishment on the party judged responsible for causing the harm (Jones, 2004). Social exchange theory assumes interpersonal relationships are governed by rules of exchange such as negotiation and reciprocity (Blau, 2017). Contacting employees outside work hours for work-related purposes can lead to involuntary “covert overtime”, transgressing work-life borders, infringing on rights, and breaching psychological contracts, consequently triggering negative reciprocity beliefs (Jensen et al., 2010). Empirical evidence shows those with negative reciprocity norms have higher retaliatory motivation (Eisenberger et al., 2004), and this leads to counterproductive work behaviors as revenge against supervisors and organizations (Glomb & Liao, 2003; H. Liao et al., 2004). In Chinese society, where hierarchical power cultures prevail and collectivist values dominate, employees are likely to respond to perceived injustice through covert behaviors (Mannan & Kashif, 2019), such as working on personal matters at worktime or gossiping with customers at work time. Therefore, this study predicts that WCB will lead to time theft behavior by triggering employees’ revenge motives.

Additionally, this study investigated the conditions under which employee theft behaviors triggered by WCB may be amplified or diminished. Existing research indicates that positive personal relationships between leaders and subordinates can enhance tolerance to work-related aggression (McLarty et al., 2021), potentially reshaping reciprocal dynamics and outcomes. Leader–member exchange (LMX) assesses the quality of the working relationship between employees and their leaders within an organization (Cogliser & Brigham, 2004). It differentiates between in-group and out-group members based on their relational quality with leaders (Berg et al., 2017). Employees in in-groups typically enjoy a closer relationship with their leaders, garnering more support and understanding, whereas out-group members receive less support and understanding and thus may perceive greater unfairness from WCB (Berg et al., 2017; Breidenthal et al., 2020). High LMX employees, perceiving significant injustice from such behaviors, are more likely to anticipate organizational recompense for their unmet commitments in the future (Shih & Lin, 2014), which can significantly mitigate their perceived injustice. This expectation and sense of obligation reduce the likelihood of developing motives for revenge. Conversely, employees with low LMX show less perspective-taking and reduced tolerance (Shih & Lin, 2014). As a result, they may develop stronger motives for revenge, leading to increased instances of time theft. Therefore, this paper predicted that LMX moderates the indirect relationship between WCB and employee time theft, with this indirect relationship becoming stronger when LMX is low.

This research advances the literature in several ways. First, it fills a gap in the literature by broadening the examination of WCB to include its consequences, such as employee time theft, a common form of workplace deviance encompassing five dimensions. Second, applying social exchange theory, the study elucidates the “black box” of the mechanisms through which WCB influences employee time theft via revenge motive, thereby addressing a gap in the literature. Third, the research enhances the understanding of the boundary conditions associated with WCB by investigating the impact of LMX. The inclusion of LMX provides a new boundary condition concerning the binary relationship between leaders and subordinates and helps complete the framework of WCB.

The remainder of this paper is structured as follows. Section 2 presents the literature review, hypotheses, and conceptual model. Section 3 outlines the research methodology. Section 4 examines the results of the measurement model and hypotheses testing. Section 5 concludes the paper and offers recommendations.

## 2. Literature Review, Hypotheses, and Conceptual Model

### 2.1. WCB

The pervasive integration of wireless technologies has precipitated a new paradigm in which constant connectivity permeates the daily operations of both individuals and organizations, thereby diminishing the clear demarcation between professional and personal time (Hassan, 2003; Kaufman-Scarborough, 2006). WCB encompasses the activities of individual employees who engage in work-related tasks during non-working hours and across various locations using mobile electronic devices, all while simultaneously managing multiple life and work roles (Brock et al., 2013). From the viewpoint of organizational leadership, the use of devices that enable wireless connectivity is believed to enhance collaborative efforts by overcoming the constraints of time and space, thus augmenting productivity (Lyytinen & Yoo, 2002). Conversely, from the employees’ viewpoint, the clear distinctions between working hours and non-working hours are increasingly eroding. In the absence of these boundaries, employees may find themselves connected to work at any time and in any place. This persistent connectivity may leave them feeling perpetually “on call” (Tarafdar et al., 2007).

WCB is characterized by unpredictability and interruption potential (Cavazotte et al., 2014). Unpredictability means employees can be informed of work issues at any time and have to expend energy to address them, disrupting their ability to leave work issues behind (Cavazotte et al., 2014). Interruptions mean work needs to interrupt personal time, such as receiving work calls at family events or in leisure and having to interrupt activities to address them (Cavazotte et al., 2014). Empirical evidence suggests that WCB with these characteristics has a negative impact on employees (Ter Hoeven et al., 2016), such as poor quality of sleep (Arlinghaus & Nachreiner, 2013), increased turnover intentions (Rasulova & Tanova, 2025), decreased job performance (Fan et al., 2024), emotional exhaustion (Y. Hu et al., 2024), and reduced job satisfaction (Li et al., 2025).

### 2.2. WCB and Employee Time Theft

Employee time theft exists in most industries (Martin et al., 2010). It involves willful failure to report and account accurately for one’s time and, in return, receive payment from the organization (Harold et al., 2022). It manifests in various forms, including unauthorized break times, excessive socialization, performing non-work-related tasks at work hours, willful reduction in work efficiency, and falsifying work hours (B. Hu et al., 2023). Empirical studies have shown that leaders aggressive humor (L. Zhao et al., 2025), careerist orientation (C. Liao et al., 2024), and pay transparency among higher-salaried coworkers (P. Zhao et al., 2025) tend to increase employee time theft, whereas socially responsible HR practices (Lv et al., 2024) and leaders’ developmental feedback (Z. Wang et al., 2024) help reduce such behavior. As a workplace deviance, time theft attracts enormous costs, including loss of productivity, increased operating costs (Schmitt et al., 2003), and work-related stress (Neuman & Baron, 1998; Penney & Spector, 2005). Because of its prevalence and negative impacts on the organization, it is essential for organizations to recognize why time theft exists and how it can be avoided.

This study suggests that WCB may lead to employee time theft. As noted, this behavior is unpredictable and frequently interrupts (Cavazotte et al., 2014). After formal work hours, employees continue to expend energy to monitor work-related messages. Even if they do not immediately address these issues, their attention shifts back to work upon receiving a notification, considering whether to resolve it then or later (Cavazotte et al., 2014). This dynamic hinders employees’ ability to psychologically disengage from work and recuperate during non-working hours (Y. Hu et al., 2024; Jarvenpaa & Lang, 2005), and the resulting excessive workload also diminishes their well-being (Ter Hoeven et al., 2016). Furthermore, the efforts involved in this “invisible workload” often lack proper compensation, leading employees to perceive inequity due to constant “obligatory labor” (Bennett & Robinson, 2000). To counter this perceived inequity, employees may not directly retaliate against their supervisors but might seek alternative methods to express their frustration and dissatisfaction by reducing their organizational contributions (Lam & Xu, 2019). These methods can include employee time theft, such as extending break times and managing personal messages during work hours.

**Hypothesis 1.**  *WCB is positively correlated with employee time theft.*

### 2.3. The Mediating Role of Revenge Motive

Revenge motive refers to an intention by a victim to harm or punish the person who inflicted an injury (Jones, 2004). This intention can be toward direct supervisors or the organization (Jones, 2004). Empirical evidence confirms that revenge motives are typically brought about by perceived injustice and unfairness (Jones, 2004) and are connected to various workplace deviant behaviors (Robinson & Greenberg, 1998). Employees feel unfairness and create revenge intentions before acting in destructive behaviors, and this confirms that such behaviors are preplanned (Robinson & Greenberg, 1998). Hence, the antecedents of revenge motives should be examined.

The present study applied social exchange theory in positing that WCB can activate employees’ revenge motives and thus lead to time theft. Social exchange theory holds that interpersonal relations are governed by some rules of exchange, including reciprocity and norms of negotiation (Blau, 2017). These relations are either positive or negative (Choi et al., 2019). Organizational behaviors involving calling employees outside work hours for work-related issues or work include the possibility of creating a sense of “implicit overtime”, where workers involuntarily work, perceiving an infringement on their personal space and rights. This infringement will most likely elicit negative reciprocity beliefs (Jensen et al., 2010). Empirical evidence suggests that when individuals become targets of abuse, those constrained by negative reciprocity are likely to develop strong revenge motivations (Barclay et al., 2014; Eisenberger et al., 2004). This study contends that such revenge motivations are likely to emerge in employees subjected to widespread WCB.

Conversely, this paper suggests that induced revenge motives likely result in employee time theft. Social exchange theory posits that the exchange process can prompt specific behaviors (Homans, 1974). When employees develop revenge motives due to perceived unfair treatment, this motivation may compromise their adherence to moral standards, leading them to engage in counterproductive work behaviors as retaliation against supervisors and the organization (Glomb & Liao, 2003; H. Liao et al., 2004). These behaviors could manifest as direct retaliation; however, studies have shown that in collectivist Asian cultures, which respect hierarchical relationships and where authority figures are revered, employees may engage in more subtle forms of workplace deviance, such as personal activities during work or prolonged breaks, thus committing what can be termed as employee time theft (Faldetta, 2021; Mannan & Kashif, 2019).

**Hypothesis 2.**  *Revenge motive mediates the relationship between WCB and employee time theft.*

### 2.4. The Moderating Role of LMX

LMX serves as a crucial metric for assessing the dynamics of relationships between employees and their leaders within organizational ecosystems (Cogliser & Brigham, 2004). Subordinates who experience high-quality LMX are regarded as integral members, benefiting from continuous emotional support, increased autonomy, enhanced knowledge acquisition, and elevated trust from their leaders (Berg et al., 2017; Breidenthal et al., 2020). Prior research has validated that employees who cultivate high-quality LMX tend to possess a more empathetic understanding of organizational policies and exhibit greater tolerance (Shih & Lin, 2014). These individuals typically exhibit lower susceptibility to envy and possess greater psychological capital (Gupta et al., 2025; Lim et al., 2024).

Thus, this study proposes that WCB is likely to incite stronger revenge motives in individuals with low LMX. When employees face WCB without corresponding rewards, they are prone to feel intense unfairness. Conversely, employees with high LMX often justify the organization’s failures, believing they might receive future compensation (Shih & Lin, 2014), which lessens their sense of unfairness. This understanding and sense of obligation toward the organization make them less susceptible to revenge motives. Additionally, previous research indicates that employees with high LMX may “repay” their leaders by expanding their role definitions (i.e., engaging in organizational citizenship behaviors) (Hofmann et al., 2003). Consequently, employees with high LMX may perceive their work connectivity as a form of role expansion, making them less likely to trigger revenge motives against their leaders. In contrast, employees with weak LMX may lack empathy, display lower tolerance and perceive their invisible overtime behaviors as an extension of their role. Thereby experiencing heightened perceptions of injustice and more intense revenge motivations. Therefore, when employees with low LMX experience WCB, their revenge motives are likely to be stronger than those of employees with high LMX.

**Hypothesis 3.**  *LMX moderates the relationship between WCB and revenge motive such that the positive correlation becomes stronger with lower levels of LMX.*

This analysis has led to the construction of a conceptual framework where the revenge motive serves as a mechanism through which WCB translates into employee time theft, with the quality of LMX acting as a moderator in this dynamic. We further suggest that that a lower quality of LMX intensifies the indirect impact of WCB on time theft via revenge motives.

**Hypothesis 4.**  *LMX moderates the indirect association between WCB and employee time theft through revenge motive, enhancing this indirect relationship when LMX is lower.*

**Figure 1:**
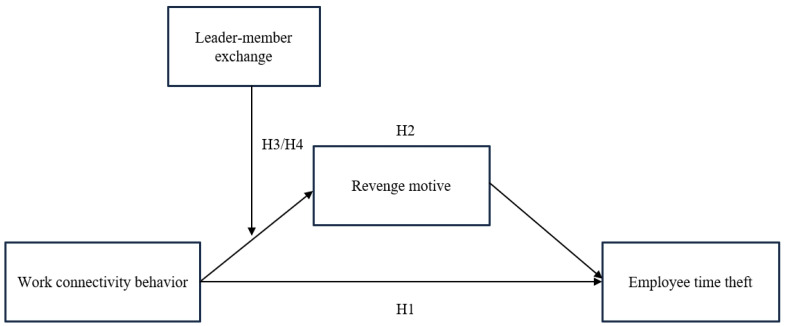
Conceptual model of this research.

Figure 1 depicts the theoretical model of this work. WCB serves as the independent variable, employee time theft as the dependent variable, and revenge motivation as the mediating variable. LMX functions as a moderating variable that both negatively moderates the relationship between WCB and revenge motivation and indirectly attenuates the WCB-time theft relationship through this mediated pathway.

## 3. Methods

### 3.1. Respondents and Procedures

The research utilized the Credamo online research platform for sample matching and data collection, involving employees from diverse industries, including information technology, internet services, finance, real estate, and e-commerce, primarily across Fujian, Guangdong, Beijing, and Shanghai. These regions were selected due to their high concentration of businesses and advanced economic activity. Additionally, mobile communication plays a critical role in the work environments of the chosen industries, ensuring the representativeness of our sample. To enhance the quality of the questionnaire, we incorporated screening questions and articulated the research objectives and risk assurances on the first page of the survey. Participants were clearly informed that their responses would remain anonymous and be utilized solely for academic purposes. Each participant who passed the quality assessment of the questionnaire received a reward of 5 RMB. A total of 360 questionnaires were distributed, and after applying the screening question and excluding those with unchanged responses, we obtained 330 valid samples.

As shown in Table 1, among the 330 valid responses, females comprised 55.5% of the sample, and approximately 71.6% of respondents were aged between 26 and 35 years. Moreover, 87.0% possessed at least a bachelor’s degree. Those with work experience ranging from three to ten years comprised 62.5% of the sample. Ordinary employees made up 50.6%, while frontline managers accounted for 26.7%. Participants preferring role integration preferred constituted 37.6%, and those reporting satisfactory sleep quality formed 69.7%.

### 3.2. Measurements

For this study, we utilized established scales widely acknowledged in academic research. To ensure linguistic and contextual accuracy, a translation and back-translation process was executed (Brislin, 1980). A pilot survey among a small group of employees preceded the formal dissemination of the questionnaires. Feedback from this group and recommendations from two subject matter experts led to slight adjustments in individual survey items to enhance the clarity and readability of the questionnaire (Schaffer & Riordan, 2003). Except for control variables, responses were gauged using a 5-point Likert scale.

WCB. This dimension of WCB was assessed using a 5-item scale developed by Büchler et al. (2020), with an example item being the following: “Through my mobile devices, I am continuously accessible to my supervisor, colleagues, or clients outside of working hours”. The reliability of this scale was recorded at 0.85.

Revenge motive. Researchers used four items from Hung et al. (2009). Two items are used to measure employees’ revenge motives toward their direct supervisors, one example being the following: “If my supervisor treats me poorly, revenging in some way makes me feel good”. The other two items are used to measure employees’ revenge motives toward the organization, one example being the following: “If my company treats me poorly, the satisfaction of ‘revenge’ outweighs the risk of being caught”. The overall reliability of the four items was 0.91.

LMX. This relationship was quantified using a 7-item scale from Graen and Uhl-Bien (1995), with an illustrative item being the following: “My working relationship with my supervisor is very good”. The reliability here was 0.90.

Employee time theft. This was assessed through a 15-item scale by Harold et al. (2022), which categorizes the behavior into five sub-dimensions: unsanctioned breaks, falsifying work hours, manipulating the speed of work, excessive socialization, and spending time on non-work tasks. One example is the following: “I intentionally reported that I worked more hours than I actually worked”. The reliabilities of the five dimensions are 0.76, 0.81, 0.79, 0.84, and 0.87. The second-order factor model exhibited a robust fit (*χ*^2^(80) = 244.98, CFI = 0.96, TLI = 0.94, RMSEA = 0.08). The reliability after aggregating the five sub-dimensions is 0.95.

Control variables. To enhance the rigor of this study, numerous control variables were incorporated, including demographic factors like gender, age, educational level, years of professional experience, and job hierarchy (Chen et al., 2022; Hung et al., 2009; C. Liao et al., 2024; Penney & Spector, 2005). Furthermore, variables such as sleep quality and personal preferences for role segmentation, known to influence employee time theft, were also included in the analysis (Olson-Buchanan & Boswell, 2006; Park et al., 2011; Senarathne Tennakoon et al., 2013; Wagner et al., 2012).

## 4. Results

### 4.1. Common Method Issues

Since the measures relied on self-reported data from the same time period, they may be subject to common method bias (Podsakoff et al., 2003). An unrotated principal component analysis using Harman’s single-factor test revealed that the primary component accounted for only 34.83% of the variance—well below the critical 50% threshold (Podsakoff et al., 2012)—indicating that the impact of common method bias is very limited. Additionally, a confirmatory factor analysis testing a single-factor model yielded unsatisfactory fit indices (*χ*^2^(189) = 3774.95, CFI = 0.33, TLI = 0.26, RMSEA = 0.23). Together, these results reinforce the robustness of our findings against common method bias.

### 4.2. Validity Test

Prior to hypothesis testing, the integrity of the measurement constructs was rigorously evaluated. As detailed in Table 2, a four-factor model provided a superior fit to the data (*χ*^2^(183) = 421.38, CFI = 0.95, TLI = 0.94, RMSEA = 0.06). All factor loadings exceeded 0.50 and were statistically significant, thereby confirming the discriminant validity of the constructs. The average variance extracted (AVE) for the constructs were 0.54, 0.56, 0.72, and 0.80, respectively, exceeding the recommended threshold of 0.50 (Hair et al., 1995) and indicating good convergent validity.

### 4.3. Descriptive Statistics

As shown in Table 3, significant positive correlations were identified between WCB and revenge motive (*r* = 0.21, *p* < 0.01), as well as between WCB and employee time theft (*r* = 0.11, *p* < 0.05). Moreover, a strong positive correlation was observed between revenge motive and employee time theft (*r* = 0.54, *p* < 0.01). The square root of the AVE for each construct was greater than its correlations with other constructs, further supporting discriminant validity.

### 4.4. Hypothesis Testing

Hierarchical multiple regression analyses were conducted to test the proposed hypotheses. As indicated in Table 4, WCB was a significant predictor of employee time theft (*β* = 0.15, *p* < 0.05, M6), thereby supporting Hypothesis 1.

Furthermore, WCB significantly predicted employees’ revenge motive (*β* = 0.31, *p* < 0.01, M2), and revenge motive, in turn, significantly predicted employee time theft (*β* = 0.31, *p* < 0.01, M7). When revenge motive was included in the regression model, the direct effect of WCB on employee time theft became non-significant (*β* = 0.06, n.s., M8), while revenge motive maintained a significant predictive effect (*β* = 0.30, *p* < 0.01, M8). The PROCESS macro (Hayes, 2015) confirmed the mediation effect, showing significant conditional effects (indirect effect = 0.09, *SE* = 0.03, 95% CI = [0.0448, 0.1422]), thereby supporting Hypothesis 2.

**Figure 2:**
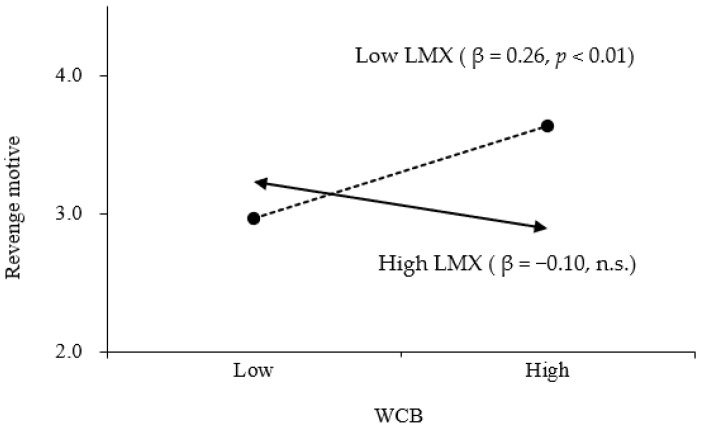
The interactive effect of WCB and revenge motive.

To examine Hypothesis 3, interaction terms were constructed using centered values of WCB and LMX. The analysis revealed that the interaction term had a significant negative effect on the revenge motive (*β* = −0.24, *p* < 0.01, M4). As shown in Figure 2. Regarding the interactive effect of WCB and revenge motive, the positive relationship between WCB and revenge motive was stronger for employees with low LMX (*β* = 0.26, *p* < 0.01) compared with those with high LMX, for whom the relationship was not statistically significant (*β* = −0.10, n.s.), supporting Hypothesis 3.

Furthermore, the conditional mediation effect was calculated using the PROCESS macro. As shown in Table 5, for employees with low LMX, the indirect influence of WCB on employee time theft was more substantial (conditional indirect effect = 0.08, *SE* = 0.03, 95% CI = [0.0246, 0.1314]), in contrast to its insignificance for those with high LMX (conditional indirect effect = −0.30, *SE* = 0.05, 95% CI = [−0.1288, 0.0518]). The disparity between these effects was statistically significant (Δconditional indirect effect = −0.11, *SE* = 0.03, 95% CI = [−0.1826, −0.0490]), supporting Hypothesis 4.

## 5. Discussion and Conclusions

### 5.1. Discussion

Based on social exchange theory, we developed a research design to confirm a moderated mediation framework. The results revealed four key findings: (1) WCB showed a significant positive correlation with employee time theft; (2) revenge motive fully mediated the WCB-time theft relationship; (3) LMX negatively moderated the WCB-revenge motive link with the effect being stronger when LMX is low; (4) LMX also moderated the indirect relationship between WCB and time theft through revenge motivative. Thus, Hypotheses 1 through 4 were fully supported.

### 5.2. Theoretical Contribution

Kundro et al. (2023) found that work detachment among high-performance-pressure employees can induce next-morning shame and subsequent cheating behavior. Following this line of reasoning, this study contributes to the advancement of work connectivity behavior in several important ways. Firstly, this study extends the literature on WCB by conceptualizing employee time theft encompassing five dimensions as a new outcome. Previous literature concentrated on the negative impacts of such activities (Dettmers et al., 2016) on employees’ health (Sonnentag & Niessen, 2020) and job satisfaction (Cheng et al., 2022). While recent research has begun to explore their effects on work–family balance (Dong et al., 2022; He et al., 2023; Y. Hu et al., 2024; Liu et al., 2024; Y. Yang et al., 2022), less work has been performed on direct work-related consequences, such as employee time theft, an insidious but damaging type of workplace deviance (Neuman & Baron, 1998; Penney & Spector, 2005). Therefore, this study significantly extends the scope of WCB implications.

Secondly, this study extends the inherent channels of WCB by adopting a novel perspective. With the introduction of social exchange theory, this study argues that perceived injustice in WCB can activate employees’ revenge motives and consequently lead to employee time theft. Previous research has revealed mediating mechanisms of ego depletion (He et al., 2023), emotional exhaustion (Y. Yang et al., 2023), work–family conflict (Liu et al., 2024), and psychological detachment (F. Wang et al., 2023). This article identifies a different mediating mechanism of revenge motive, which reveals the desire motivations that arise from a reciprocity perspective when employees perceive unfairness in the workplace. This retaliatory motivation based on perceived injustice provides a significant theoretical extension in explaining employee time theft motivation in the context of overtime work.

Lastly, this study identifies new boundary conditions for WCB. The findings indicate that LMX can mitigate employees’ revenge motives related to WCB, thereby indirectly reducing instances of employee time theft. Previous research primarily focused on individual traits as moderating variables, such as leader workaholism (Dong et al., 2022), employee work-family segmentation preferences (Y. Hu et al., 2024), and employee intrinsic motivation (R. Wang et al., 2023), or the perspective of family support (He et al., 2023). This boundary condition moves beyond previous considerations by introducing a new dimension based on the perceived relationship between employees and their leaders. This approach enriches the literature by providing a more comprehensive framework for understanding WCB.

### 5.3. Practical Implications

Firstly, this research encourages business managers to adopt an equal-minded approach towards WCB. It reveals that while companies may hope to benefit from WCB beyond official hours—such as increased productivity or enhancing employee autonomy—they may inadvertently reward detrimental behaviors like time theft. This prompts managers to reconsider an “efficiency-first” strategy (Y. Yang et al., 2023). This strategy can unintentionally produce “hidden strikes” by workers. In order to escape perceptions of unfairness, companies have to develop scientifically founded work systems and remuneration systems that recognize and remunerate the workload performed outside working hours.

Secondly, by incorporating the revenge motive in our study model, we explored its mediating role between WCB and employee theft of work hours. This information provides insight into the mechanistic effects of the negative impacts of WCB and offers an important case for in-depth examination. Managers are encouraged to engage in regular in-depth interactions with workers in order to know their true sentiments and opinions and to avoid the magnification of negative effects. In addition, the creation and utilization of anonymous feedback lines in the organization can allow managers to hear workers’ internal thoughts and employees to remain anonymous, and this helps to stem the development of revenge motives.

Thirdly, examining the moderating role of LMX sharpens the focus of enterprises on the relationship between leaders and employees. A strong leader–member relationship not only reduces employees’ revenge motives but also alleviates employee time theft stemming from WCB. This suggests that managers can shape interpersonal relationships between leaders and employees by regularly organizing team-building activities and daily behavioral interventions. For instance, organizations can foster interactions between leaders and employees through team dinners or recreational activities, enhancing mutual understanding of each other’s needs and expectations. At the same time, leaders should exhibit a positive attitude of listening and responding to bolster employees’ sense of belonging and participation. Such attention and investment are likely to lead to higher employee satisfaction and stronger team cooperation over time, ultimately boosting the overall performance and competitiveness of the organization (Chan & Mak, 2012).

### 5.4. Limitations and Future Research

One limitation is that the participants in this study were primarily under 40 years of age, representing a fairly young segment of the labor force. Consequently, the results apply mainly to the younger labor demographic, while the generalizability to older and more experienced workforce segments needs further exploration. Future research should thus include more broadly representative groups to enhance validation.

Furthermore, all variables in this study were gauged through self-report measures. While this approach is often considered optimal in organizational research, participants may feel pressured to provide socially desirable responses. Furthermore, self-report methods are inherently susceptible to common method bias. However, in this study, potential biases were minimized by ensuring strict confidentiality and anonymity for all participants, with data used solely for academic purposes. Future research could consider employing longitudinal surveys to further reduce potential biases. If conditions permit, it is advisable to use specific measuring instruments in subsequent studies to mitigate these effects.

Lastly, it is also critical to acknowledge that the scope of this study is confined to China, a nation that highly values harmonious social interactions and adheres to well-established social norms (K. S. Yang, 1995). In the collectivist setting of Asia, there is a prevalent acceptance of leaders’ authority among employees (Mannan & Kashif, 2019). When faced with exploitation by authority figures, this compliance may prevent individuals from directly resisting and instead lead them to seek indirect revenge through acts such as time theft. Responses in this context may differ significantly from those in Western countries, where the culture places a greater emphasis on individualism (Peterson, 2003). Therefore, while this study confirms that WCB can lead to employee time theft through revenge motive, this mechanism may be influenced by the Chinese context, and its applicability in other cultural settings still requires broader research to validate the universality of the findings.

## Figures and Tables

**Table 1:** The distribution characteristics of the sample.

Characteristics	Type	Frequency	Percent (%)
Gender	Male	147	44.5
	Female	183	55.5
Age(year)	Less than 25	52	15.8
	26–30	119	36.1
	31–35	117	35.5
	36–45	32	9.7
	More than 45	10	3
Education	High school or below	8	2.4
	Associate degree	32	9.7
	Bachelor’s degree	247	74.8
	Master’s degree or above	43	13
Years of work experience	Less than 1	25	7.6
	1–3	80	24.2
	3–5	86	26.1
	5–10	120	36.4
	More than 10	19	5.8
Job position level	Ordinary staff	167	50.6
	Frontline management	88	26.7
	Middle management	57	17.3
	Top management	18	5.5
Sleep quality	Poor	15	4.5
	Average	85	25.8
	Good	230	69.7
Role integration preference	Dislike	206	62.4
	Like	124	37.6

**Table 2:** CFA results.

Model	*χ* ^2^	*df*	CFI	TLI	RMSEA
Four-factor model:	421.38	183	0.95	0.94	0.06
Three-factor model 1:	821.70	186	0.87	0.85	0.10
Combining WCB and LMX					
Three-factor model 2:	1335.39	186	0.76	0.73	0.14
Combining revenge motive and employee time theft					
Two-factor model:	1418.30	188	0.73	0.71	0.14
Combining WCB, LMX and revenge motive					
One-factor model:	3774.95	189	0.33	0.26	0.23
Combining all variables					

Notes: *N* = 330; *χ*^2^ = chi-square; *df* = degrees of freedom; CFI = comparative fit index; TLI = Tucker–Lewis index; RMSEA = root-mean-square error of approximation. Employee time theft was simplified into five items; WCB = work connectivity behavior; LMX = leader–member exchange.

**Table 3:** Descriptive statistics and correlations.

	1	2	3	4	5	6	7	8	9	10	11
1. Gender	-										
2. Age	−0.04	-									
3. Education	−0.05	−0.09	-								
4. Years of work experience	−0.04	0.67 **	0.04	-							
5. Job position level	−0.11	0.35 **	0.17 **	0.35 **	-						
6. Sleep quality	0.00	−0.04	0.02	0.16 **	0.01	-					
7. Role integration preference	0.05	−0.05	0.12 *	0.17 **	0.14 *	0.20 **	-				
8. WCB	0.21 **	−0.05	−0.04	−0.06	0.13 *	−0.07	0.20 **	(0.73)			
9. LMX	0.14 **	0.09	−0.01	0.15 **	0.19 **	0.23 **	0.36 **	0.54 **	(0.75)		
10. Revenge motive	0.09	−0.23 **	−0.05	−0.35 **	−0.13 *	−0.29 **	−0.19 **	0.21 **	−0.05	(0.85)	
11. Employee time theft	−0.01	−0.22 **	−0.06	−0.38 **	−0.17 **	−0.27 **	−0.26 **	0.11 *	−0.23 **	0.54 **	(0.89)
Mean	0.55	2.48	2.98	3.08	1.78	2.65	0.38	4.16	3.86	3.18	2.43
SD	0.50	0.97	0.57	10.07	0.92	0.57	0.49	0.70	0.77	1.08	0.78

Notes: *N* = 330; bracketed value on the diagonal are the square root of the average variance extracted value of each scale; ** *p* < 0.01 (two-tailed), * *p* < 0.05 (two-tailed); WCB = work connectivity behavior; LMX = leader–member exchange.

**Table 4:** Regression analysis results.

	Revenge Motive				Employee Time Theft			
	M1	M2	M3	M4	M5	M6	M7	M8
Control variables								
Gender	0.17	0.08	0.09	0.06	−0.02	−0.07	−0.08	−0.09
Age	−0.12	−0.11	−0.11	−0.11	−0.04	−0.03	0.00	0.00
Education	−0.06	−0.03	−0.03	−0.05	−0.03	−0.02	−0.02	−0.01
Years of work experience	−0.22 **	−0.20 **	−0.20 **	−0.15 *	−0.20 **	−0.19 **	−0.13	−0.13 **
Job position level	0.02	−0.02	−0.02	−0.05	−0.03	−0.05	−0.04	−0.05
Sleep quality	−0.44 **	−0.41 **	−0.40 **	−0.35 **	−0.26 **	−0.25 **	−0.13	−0.12
Role integration preference	−0.26 *	−0.35 **	−0.34 **	−0.31*	−0.27 **	−0.31 **	−0.19	−0.21 *
Independent variable								
WCB		0.31 **	0.32 **	0.08		0.15 *		0.06
Mediator								
Revenge motive							0.31 **	0.30 **
Moderator								
LMX			−0.03	−0.11				
Interaction								
WCB × LMX				−0.24 **				
*R* ^2^	0.20	0.23	0.23	0.26	0.21	0.23	0.36	0.36
Δ*R*^2^	0.20	0.04	0.00	0.03	0.21	0.02	0.15	0.13
*F*	11.23 **	12.04 **	10.68 **	11.16 **	12.46 **	11.91 **	22.55 **	20.17 **

Notes: *N* = 330; ** *p* < 0.01 (two-tailed), * *p* < 0.05 (two-tailed); WCB = work connectivity behavior; LMX = leader–member exchange.

**Table 5:** Conditional indirect effects of WCB on revenge motive (at ±1 SD of LMX).

	Effect (*SE*)	LLCI	ULCI
Low LMX (−1 SD)	0.08 (0.03)	0.0246	0.1314
High LMX (+1 SD)	−0.03 (0.05)	−0.1288	0.0518
Difference	−0.11 (0.03)	−0.1826	−0.0490

Notes: *N* = 330; Bootstrap sample size = 5000; Bootstrapped estimates for standard errors are presented in parentheses; LLCI = Lower level of the 95% confidence interval; ULCI = Upper level of 95% confidence interval; WCB = work connectivity behavior; LMX = leader–member exchange.

## Data Availability

The datasets analyzed in this study are available from the corresponding author upon reasonable request.

## Abbreviations

The following abbreviations are used in this manuscript:

WCB	Work connectivity behavior
LMX	Leader–member exchange
